# Induction of granzyme B expression in T-cell receptor/CD28-stimulated human regulatory T cells is suppressed by inhibitors of the PI3K-mTOR pathway

**DOI:** 10.1186/1471-2172-10-59

**Published:** 2009-11-22

**Authors:** Olga V Efimova, Todd W Kelley

**Affiliations:** 1 Department of Pathology, University of Utah, Salt Lake City, Utah, USA; 2 ARUP Institute for Clinical and Experimental Pathology, ARUP Laboratories, Salt Lake City, Utah, USA

## Abstract

**Background:**

Regulatory T cells (Tregs) can employ a cell contact- and granzyme B-dependent mechanism to mediate suppression of bystander T and B cells. Murine studies indicate that granzyme B is involved in the Treg-mediated suppression of anti-tumor immunity in the tumor microenvironment and in the Treg-mediated maintenance of allograft survival. In spite of its central importance, a detailed study of granzyme B expression patterns in human Tregs has not been performed.

**Results:**

Our data demonstrated that natural Tregs freshly isolated from the peripheral blood of normal adults lacked granzyme B expression. Tregs subjected to prolonged TCR and CD28 triggering, in the presence of IL-2, expressed high levels of granzyme B but CD3 stimulation alone or IL-2 treatment alone failed to induce granzyme B. Treatment of Tregs with the mammalian target of rapamycin (mTOR) inhibitor, rapamycin or the PI3 kinase (PI3K) inhibitor LY294002 markedly suppressed granzyme B expression. However, neither rapamycin, as previously reported by others, nor LY294002 inhibited Treg proliferation or induced significant cell death in TCR/CD28/IL-2 stimulated cells. The proliferation rate of Tregs was markedly higher than that of CD4+ conventional T cells in the setting of rapamycin treatment. Tregs expanded by CD3/CD28/IL-2 stimulation without rapamycin demonstrated increased *in vitro *cytotoxic activity compared to Tregs expanded in the presence of rapamycin in both short term (6 hours) and long term (48 hours) cytotoxicity assays.

**Conclusion:**

TCR/CD28 mediated activation of the PI3K-mTOR pathway is important for granyzme B expression but not proliferation in regulatory T cells. These findings may indicate that suppressive mechanisms other than granzyme B are utilized by rapamycin-expanded Tregs.

## Background

Thymus-derived regulatory T cells (Tregs), or natural Tregs (nTregs), suppress the proliferation of bystander T cells through CTLA-4, IL-10 or secreted or membrane-bound forms of TGF-β1 [1]. Granzyme B-mediated suppressive mechanisms have also been identified and result in the selective killing of antigen presenting B cells [2] and bystander effector T cells [3]. In the tumor microenvironment, granzyme B is important for Treg-mediated suppression of tumor clearance [4]. Recent evidence also demonstrates a role for Treg-specific granzyme B expression in the initiation and maintenance of allograft tolerance [5]. Thus, granzyme B mediated induction of apoptosis in target cells represents an additional suppressive mechanism utilized by Tregs. Many of the aforementioned studies of granzyme B were performed in murine models utilizing pre-activated Tregs. Preactivation with sustained T-cell antigen receptor (TCR) and CD28 co-receptor stimulation in the presence of interleukin-2 (IL-2) appears to enhance suppressive abilities over those of freshly isolated nTregs [6]. TCR/CD28/IL-2 stimulation also results in marked expansion of Tregs and is useful for the generation of sufficient numbers for adoptive transfer. Expansion may also influence the expression of granzyme B, but a detailed study of granzyme B expression patterns in fresh and expanded human *ex vivo *nTregs has not been performed.

IL-2 is essential for the development, maintenance and function of the regulatory T cell pool. High-level expression of the IL-2R alpha chain (CD25) is characteristic of Tregs although it is not specific for them as it is also expressed on activated effector T cells. Mice deficient in specific components of the IL-2-IL-2R signaling pathway suffer from severe autoimmune disease [7] and a lymphoproliferative syndrome [8,9] and lack functional Tregs [10]. Triggering of the IL-2R results in the phosphorylation of STAT5 which binds the promoter region of the *FOXP3 *gene suggesting that it has a regulatory function [11]. Further, T cell specific deletion of *STAT5 *results in reduced numbers of Tregs in mice [11]. These studies demonstrate the importance of IL-2 and the central dependence on IL-2R mediated STAT5 activation for promotion of FOXP3 expression and acquisition of a suppressive phenotype.

A previous study has shown that IL-2 alone is sufficient to induce granzyme B and lytic activity in CD8-positive T cells without TCR stimulation [12]. Natural killer (NK) cells also upregulate granzyme B in response to IL-2 alone [13,14] and transcription of *granzyme B *in primary NK cells, an NK cell line (NK92) and a T cell line (Jurkat) requires IL-2 mediated NF-κB activation [14]. Although phosphoinositide-3-kinase (PI3K) can mediate NF-κB activation in diverse cell types, neither the PI3K inhibitor LY294002 nor mitogen activated protein kinase (MAPK) pathway inhibitors suppress IL-2 stimulated granzyme B expression in NK92 NK cells [14]. Since Tregs exhibit some distinct differences in PI3K pathway signaling, including an altered pattern of AKT activation [4,15,16] and altered IL-2R signaling [17], there may be differences in the reliance on pathways upstream of NF-κB for the induction of granzyme B.

Recent clinical reports suggest that rapamycin, but not cyclosporine, preserves the peripheral CD4+, CD25+, FOXP3+ regulatory T cell pool in transplant patients [18,19]. *In vitro*, rapamycin preferentially suppresses the proliferation of mouse CD4+CD25- T cells but not CD4+CD25^bright ^nTregs under conditions of TCR/CD28 activation in the presence of IL-2 [20,21]. This is likely due to an inherent lack of dependence, by Tregs, on the mammalian target of rapamycin (mTOR) pathway for proliferation or maintenance of FOXP3 expression levels [20]. Since STAT5 activation is critical for Treg maintenance, the presence of rapamycin could theoretically enhance IL-2R mediated activation of the Janus tyrosine kinase (JAK)/STAT5 pathway thereby favoring Tregs. Therefore, rapamycin may have both *in vivo *and *in vitro *utility for increasing the relative abundance of Tregs in a physiologically heterogeneous T cell population. An *in vitro *study using human alloreactive conventional CD4+ and CD8+ T cells showed decreased granzyme B expression and decreased cytotoxic activity in rapamycin treated cells [22]. However, the effects of rapamycin on granzyme B expression in human Tregs have not previously been evaluated.

In this study we sought to clarify the signaling pathways in Tregs that are important for granzyme B expression including the necessity for TCR and CD28 activation. Furthermore, we sought to evaluate the effects of PI3K pathway inhibitors on granzyme B expression, including the clinically important mTOR inhibitor rapamycin. We found that engagement of both the TCR and CD28 was necessary for granzyme B expression. Inhibitors of the PI3K-mTOR pathway including both rapamycin and LY294002 markedly suppressed granzyme B expression but did not prevent proliferation or interfere with FOXP3 expression levels. In an *in vitro *cytotoxicity assay using a Hodgkin lymphoma cell (L428) that we have previously observed to be susceptible to Treg-mediated killing (unpublished observation), Tregs expanded in the presence of rapamycin had a significant decrease in cytotoxicity as compared to Tregs expanded without rapamycin. The finding that rapamycin suppresses granzyme B expression may indicate that rapamycin-expanded Tregs utilize suppressive pathways other than granzyme B to mediate their effects on bystander lymphocytes under physiologic conditions.

## Methods

### Flow cytometry

The following antibodies were used for the flow cytometric evaluation of T cell subsets in this study: anti-CD4 (clone RPA-T4), anti-CD25 (clone BC96), anti-CD127 (clone HCD127), anti-FOXP3 (clone 259D), all from Biolegend Inc. (San Diego, CA). Anti-granzyme B (clone GB11) was from eBioscience (San Diego, CA). For the carboxy-fluorescein diacetate, succinimidyl ester (CFSE)-based proliferation studies only, anti-FOXP3 (clone 259D/C7) was purchased from BD Biosciences (San Jose, CA). Cells were permeabilized using a commercially available fixation and permeabilization kit (FOXP3 fix/perm kit; Biolegend) per the manufacturer's instructions. Propidium iodide (PI) and annexin V-PE were from Pharmingen (San Diego, CA). CFSE (Invitrogen, Carlsbad, CA) was used at a final concentration of 1 μM. Cells were labeled for 5 minutes in phosphate buffered saline (PBS) containing CFSE at 37°C then placed in pre-warmed media for 10 minutes, washed two times and plated as indicated in the figures. Flow cytometry was performed on a FACSCanto II flow cytometer (BD Biosciences) using FACSDiva software (BD Biosciences) except for cell sorting which was performed on a FACSVantage flow cytometer (BD Biosciences). Final data analysis was performed using FloJo software (Tree Star, Inc, Ashland, OR). The statistical significance of differences in granzyme B expression and Foxp3 expression between subsets was assessed using the Student's t-test.

### Regulatory T cell isolation/enrichment

Institutional review board (IRB) approval was obtained from the University of Utah Health Sciences Center to obtain peripheral blood from normal adult volunteers. All blood donors provided informed consent prior to blood collection. Blood was collected by peripheral venipuncture in ethylenediaminetetra-acetic acid (EDTA)-coated vacutainer tubes (BD, Franklin Lakes, NJ). Peripheral blood mononuclear cells were isolated from normal donors by density gradient centrifugation using Ficol-Paque density medium (GE Healthcare, Uppsala, Sweden) according to the manufacturers recommendations. Subsequently, CD4+, CD25+ Tregs were enriched using a magnetic bead-based kit (Miltenyi Biotec, Auburn CA) according to the manufacturers instructions. This technique routinely resulted in samples that were enriched in regulatory T cells to a purity of approximately 60-70% or more based on FOXP3 staining by flow cytometry. However, higher purity Tregs were required for the cytotoxicity assays. For the 6-hour cytotoxicity assays, peripheral blood nTregs were isolated based on flow cytometric sorting of the CD4+, CD25^bright ^(brightest 1/3 of CD25 positive cells) T cell fraction. For the 48-hour cytotoxicity assays, isolation based on the CD4+, CD25+, CD127-dim/negative expression pattern characteristic of Tregs was performed using a magnetic bead based kit (Miltenyi Biotec). Both methods resulted in a similar pre-expansion purity of >90% Tregs, based on flow cytometic evaluation of FOXP3 expression.

### Cell culture, stimulation, expansion and cytotoxicity

Human peripheral blood T cells were maintained in RPMI 1640 medium supplemented with 10% fetal calf serum (both from Hyclone, Logan, UT) at 37°C in 5% CO_2 _for the indicated time. Where indicated, cells were expanded with anti-CD3/anti-CD28 coated beads (Invitrogen) or anti-CD3 coated beads (Invitrogen), both at a bead:cell ratio of 4:1. Where indicated, recombinant human IL-2 (rhIL-2) at 250 IU/mL (R&D Systems, Minneapolis, MN) was also added. In some experiments, inhibitors were added to the culture 30 minutes prior to the addition of the above stimulants. These included rapamycin (Calbiochem, San Diego, CA) at 10 ng/mL or 100 ng/mL, or the PI3K inhibitor LY294002 (Cell Signaling Technology, Danvers, MA) at 1 μM or10 μM, all in dimethyl sulfoxide (DMSO; Sigma, St. Louis, MO). In some cases, freshly enriched CD4+, CD25+ T cell samples were frozen in the vapor phase of liquid nitrogen in freezing medium (10% DMSO in RPMI 1640 medium with 20% fetal calf serum) then thawed and analyzed by flow cytometry at the same time as their cultured (stimulated) counterparts. These samples are designated as "unstimulated" in the figures. Freezing in this way did not have an effect on antigen expression patterns or viability. The human Hodgkin lymphoma cell line, L428 was obtained from the German Collection of Microorganisms and Cell Cultures (Braunschweig, Germany) and maintained in RPMI medium supplemented with 10% fetal calf serum. For the cytoxocity assays, purified peripheral blood nTregs were expanded as indicated then co-cultured at a 3:1 effector:target ratio with L428 target cells, pre-labeled with CFSE in media without added stimuli. Controls consisted of L428 target cells alone or L428 target cells co-cultured with non-activated conventional CD4+ T cells (Tconv). An additional positive control for the 6-hour samples consisted of L428 cells alone cultured with 10 μM staurosporine (Sigma) to induce apoptosis. Samples were then stained with annexin-V-PE to assess early apoptosis in the 6-hour assays or for propidium iodide to assess late apoptosis in the 48-hour assays. Annexin-V binding or PI staining were then assessed in the CFSE labeled target cell population to quantify apoptosis. Significance was assessed with the Student's t test.

## Results

### Granzyme B is not expressed by freshly isolated peripheral blood CD4+, CD25+ Tregs or CD4+, CD25- conventional T cells from normal adults

**Figure 1:**
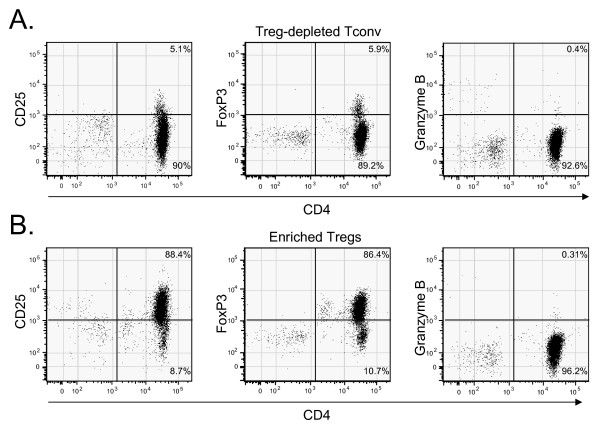
**CD4, CD25, granzyme B and FOXP3 expression patterns in freshly isolated Treg-depleted peripheral blood CD4+, CD25- conventional T cells (panel A) and enriched CD4+, CD25+ natural regulatory T cells (panel B)**. Utilizing a CD4/CD25 magnetic bead-based technique, the enriched samples of nTregs were routinely comprised of 60-70% Tregs based on FOXP3 expression. This data is representative of that seen in multiple subsequent experiments utilizing enriched Tregs. Similar results were obtained in 4 normal donors.

Tregs and Tconv were isolated from the peripheral blood of normal adults. Freshly isolated cells were evaluated for expression of CD4, CD25, FOXP3 and granzyme B by flow cytometry. FOXP3 expression was only seen in the CD25+ subset. Granzyme B expression was not detected in peripheral blood CD4+ T cells in any of the freshly isolated samples from the four normal volunteers tested. Representative dot plots showing expression of the above markers are shown in Figure 1 for the fraction depleted of Tregs (Figure 1A) and the fraction enriched in Tregs (Figure 1B).

### Expansion of ex vivo enriched human Tregs with CD3/CD28 stimulation and IL-2 induces expression of granzyme B but IL-2 alone or CD3 stimulation alone does not

**Figure 2:**
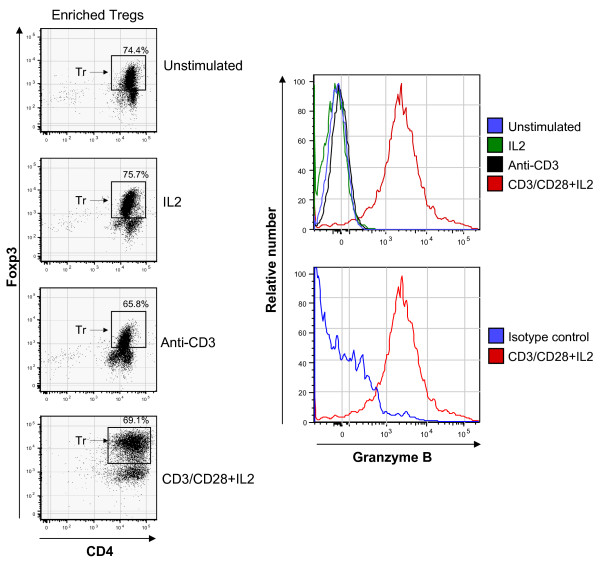
**Granzyme B expression patterns by flow cytometry in enriched Tregs freshly isolated (unstimulated) or Tregs treated with IL-2 (250 IU/mL) or anti-CD3 coated beads (4:1 ratio of beads to cells) or anti-CD3/anti-CD28 coated beads (4:1 ratio of beads to cells) plus IL-2 (250 IU/ml) for 4 days**. Granzyme B expression in the FOXP3+ gated Treg population at left (labeled Tr) is shown in histogram form at right (top right). For ease of comparison, the bottom right plot shows the level of staining of CD3/CD28/IL-2 stimulated cells with a granzyme B isotype control antibody. The data is representative of more than three experiments using multiple donors.

Tregs were enriched from peripheral blood then subjected to stimulation for 4 days *in vitro *using IL-2 alone (250 IU/mL), CD3 stimulation alone with anti-CD3 coated beads or combined CD3/CD28 stimulation with anti-CD3 and anti-CD28 coated beads with IL-2 (250 IU/mL). On day 4, samples were simultaneously stained for CD4, FOXP3 and granzyme B (or an equal concentration of isotype control antibody instead of anti-granzyme B) and evaluated by flow cytometry (Figure 2). Granzyme B was assessed in the distinct FOXP3 bright population representing Tregs (gate shown) and granzyme B staining was expressed as mean fluorescence intensity (MFI) (Figure 2). Neither IL-2 alone or anti-CD3 alone promoted an increase in granzyme B expression. However, anti-CD3/anti-CD28 coated beads plus IL-2 induced a marked increase in the level of granzyme B staining. Staining of a duplicate CD3/CD28 and IL-2 stimulated sample with anti-CD4, anti-FOXP3 and a granzyme B isotype control antibody confirms the specificity of anti-granzyme B antibody staining (Figure 2).

### Inhibition of mTOR by rapamycin and inhibition of PI3K by LY294002 suppresses granzyme B expression in TCR/CD28/IL-2 stimulated Tregs

**Figure 3:**
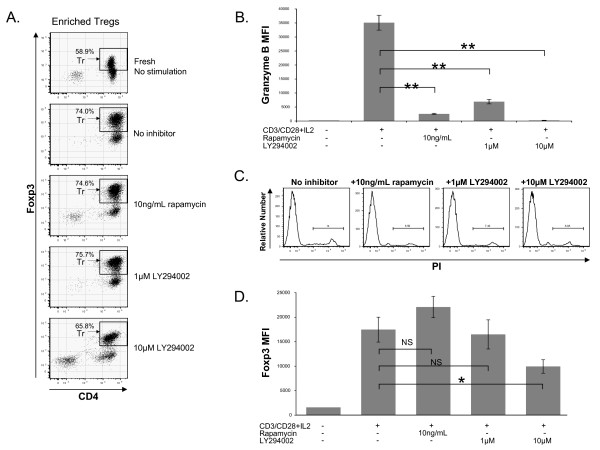
**Granzyme B expression in Tregs analyzed by flow cytometric gating on FOXP3-bright cells (panel A, population labeled Tr) without stimulation or with stimulation by CD3/CD28 beads and IL-2 for 4 days without inhibitors (labeled no inhibitor) or with rapamycin (10 ng/mL) or the PI3K inhibitor LY294002 (1 or 10 μM)**. The data is graphed as mean of the mean fluorescence intensities (MFI) of granzyme B staining in the FOXP3-bright population in triplicate samples (panel B). Statistical comparison of the indicated data sets using a t-test is shown (** denotes P < .003). In order to confirm that the decrease in granzyme B expression was not attributable to induction of cell death in the rapamycin or LY294002-treated cells, samples were stained with propidium iodide (PI) (panel C). To facilitate interpretation, we next compared FOXP3 MFIs in the samples. The level of FOXP3 expression in the gated FOXP3-bright population (see panel A) in one unstimulated sample of Tregs (panel D, far left) and in triplicate stimulated samples treated with inhibitors as indicated is shown as a bar graph (panel D). Pairwise statistical comparisons are shown (NS-not significant with P > .05; * denotes P = .02). This data is representative of more than three experiments from multiple donors.

Enriched peripheral blood nTregs were subjected to *in vitro *expansion for 4 days using CD3/CD28 beads and IL-2 (250 IU/mL) in the presence of the indicated inhibitor. In the case of rapamycin, similar conditions have previously been shown to lead to selective expansion of CD4+, CD25^bright^, FOXP3+ Tregs with retained suppressive capabilities [21]. Using flow cytometric evaluation of CD4, FOXP3 and granzyme B and gating on FOXP3-bright cells (Figure 3A), we found that PI3K inhibition resulted in a dose-dependent suppression of granzyme B expression that was complete at 10 μM LY294002 (Figure 3B). Rapamycin treatment at only 10 ng/mL also resulted in marked suppression of granzyme B expression (Figure 3B). To confirm that these findings were not simply due to the induction of cell death by the inhibitors, the cultures were evaluated for PI staining by flow cytometry (Figure 3C). There was no increase in the number of PI positive cells in any of the inhibitor treated samples as compared to samples without inhibitor treatment. Flow cytometric evaluation of FOXP3-bright cells demonstrated an activation induced increase in the level of FOXP3 expression (Figure 3A and 3D), versus that seen in freshly isolated peripheral blood Tregs in all stimulated samples, regardless of inhibitor. Evaluation of triplicate samples showed no significant suppression (or augmentation) of activation-induced enhancement of FOXP3 expression in the rapamycin or low dose LY294002 treated samples (Figure 3D). The sample treated with high dose LY294002 (10 μM) showed a slight but significant decrease in activation induced FOXP3 enhancement (Figure 3D). Given this finding, we conclude that there is no correlation between granzyme B expression and FOXP3 expression.

### CD4+, FOXP3+, CD127 negative Tregs expand preferentially in the setting of rapamycin treatment when compared with CD4+, FOXP3-, CD127+ Tconv

**Figure 4:**
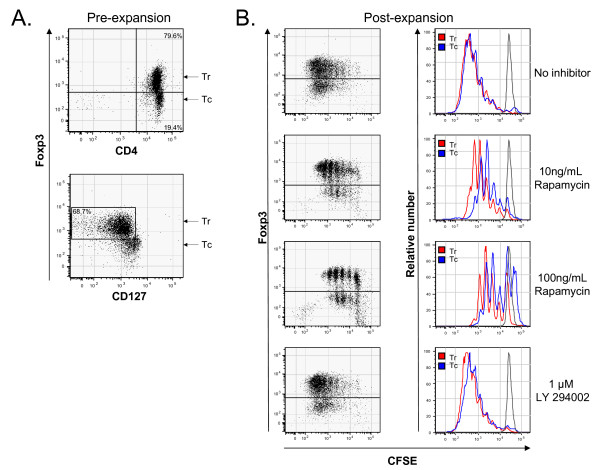
**Treg-enriched peripheral blood CD4+ T cells containing approximately 80% Tregs (Tr) and 20% Tconv (Tc) were evaluated for expression of CD4, FOXP3 and CD127 prior to expansion (panel A)**. Samples were next labeled with CFSE then expanded for six days with CD3/CD28 beads and IL-2. Subsequently, samples were simultaneously evaluated for FOXP3 expression and CFSE staining intensity by flow cytometry. Dot plots showing proliferation in the FOXP3+ and FOXP3- fractions are shown in panel B along with an overlay of histograms for CFSE staining (Tregs are shown in red and Tconv are shown in blue). As a control a CFSE-labeled but unstimulated sample is shown in black. The data is representative of three similar experiments from multiple donors.

The proliferation of Tregs and Tconv in response to the strong stimuli of CD3/CD28 beads and IL-2 was assessed in the presence of mTOR or PI3K inhibitors. Enriched peripheral blood Tregs (approximately 80% Tregs, 20% Tconv), pre-labeled with CFSE, were cultured with rapamycin (10 and 100 ng/mL) or LY294002 (1 μM) plus CD3/CD28 beads and IL-2 (250 IU/mL) for 4 days. To quantify the initial number of Tregs and Tconv in the culture, CD4, FOXP3 and CD127 levels were assessed by flow cytometry prior to expansion (Figure 4A). After expansion, single cell evaluation of CFSE staining and FOXP3 expression was performed by flow cytometry in order to separately quantify proliferation in the FOXP3+ and FOXP3- subsets. This revealed preferential expansion of Tregs in the presence of rapamycin at 10 ng/mL and 100 ng/mL but similar proliferation in the sample expanded without inhibitors and in the sample expanded in the presence of LY294002 (Figure 4B). To more rigorously compare the proliferative response of the FOXP3+ and FOXP3- fractions in the context of mTOR inhibition by rapamycin the percentage of cells in each generation was quantified (Table 1). In the setting of 10 ng/mL rapamycin, which corresponds to therapeutic human plasma levels [23], six total Treg generations and five Tconv generations could be defined by CFSE staining with 42.3% of Tregs in generations 5 and 6 versus 10.4% of Tconv. In the samples subjected to the highest rapamyin concentration (100 ng/mL) similar results were obtained although there was suppression of proliferation in both Treg and Tconv fractions (22% of Tregs in generations 4 and 5 vs 6.7% of Tconv). These findings show that under identical conditions, proliferation of Tregs is highly favored by rapamycin treatment.

**Table 1:** Percent cells in each generation, based on CFSE staining, for the rapamycin treated samples shown in Figure 4B.

Generation	CD3/CD28/IL2+10 ng/ml rapamycin		CD3/CD28/IL2+100 ng/ml rapamycin	
	
	FOXP3- (%)	FOXP3+ (%)	FOXP3- (%)	FOXP3+ (%)
**0**	7.9	3.9	22.1	23.8

**1**	9.7	5.4	17	8.3

**2**	17.5	8.4	28.1	17

**3**	28.9	14.6	26.1	28.9

**4**	25.6	25.4	6.7	19.2

**5**	10.4	28.4	- - -	2.8

**6**	- - -	13.9	- - -	- - -

**Total**	100	100	100	100

### Expansion of CD4+, FOXP3+, CD127- Tregs results in upregulation of the IL-7R to levels equivalent to those seen in FOXP3- Tconv

**Figure 5:**
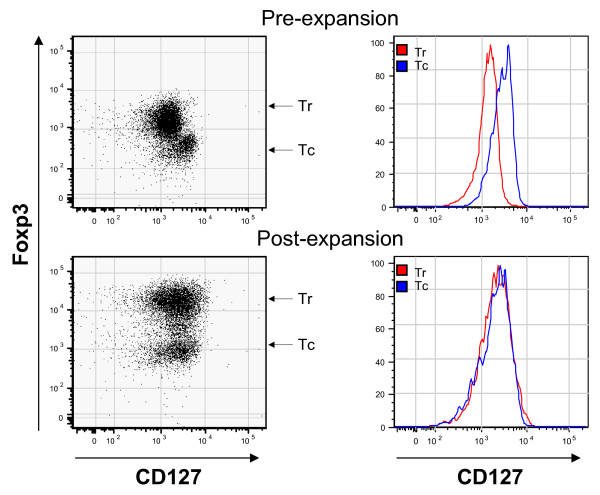
**CD4+ peripheral blood T cells, enriched for Tregs but containing a population of Tconv, were evaluated by flow cytometry for CD127 and FOXP3 prior to expansion and after 4 days of expansion with CD3/CD28 beads and IL-2**. A comparison of the level of CD127 staining is shown in the histograms (Tr = Tregs, Tc = Tconv). The data is representative of multiple experiments using different donors.

The IL-7 receptor (CD127) has been shown to be expressed at lower levels in Foxp3+ Tregs and to inversely correlate with their suppressive capacity [24]. In agreement, we found that prior to expansion, CD127 levels were indeed lower in FOXP3+ Tregs (Figure 4A/Figure 5). We wondered whether expansion with CD3/CD28 stimulation and IL-2 resulted in a retained pattern of low CD127 expression in Tregs. Interestingly, we found essentially identical CD127 levels in expanded Tregs and Tconv (Figure 5) suggesting a limited utility for CD127-based purification techniques for separating *in vitro *expanded Tregs from Tconv.

### Tregs expanded in vitro without rapamycin have increased in vitro cytotoxicity versus Tregs expanded in rapamycin

**Figure 6:**
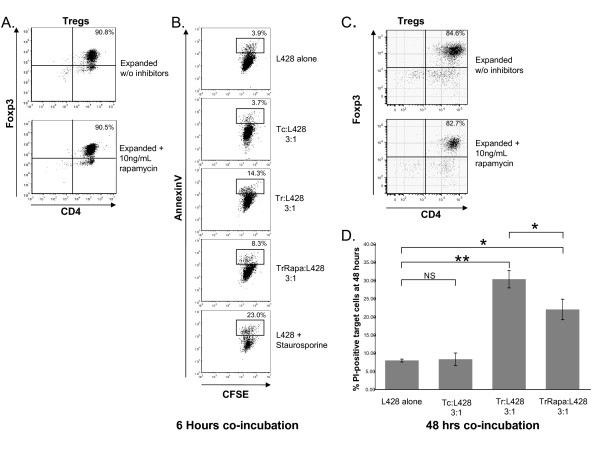
**Fresh peripheral blood nTregs were purified based on flow cytometric sorting of the CD4+, CD25-bright T cell subset (brightest 1/3 of CD25+ cells; Figure 6A/B) or by a bead based method based on a CD4+, CD25+, CD127- surface antigen expression profile (Figure C/D) then expanded for four days with CD3/CD28 beads and IL-2 with and without 10 ng/mL rapamycin**. Both isolation methods gave similar results. CD4 and FOXP3 expression were then evaluated on day 4 and showed relative purity of the resulting Treg populations (90.8% and 90.5% in panel A and 85% and 83% as indicated in panel C). Tregs were then co-cultured at a 3:1 ratio of Tregs (Tr = Tregs expanded without rapamycin; TrRapa = Tregs expanded with rapamycin) to CFSE labeled L428 target cells. Samples were plated in triplicate in media without additional stimuli or inhibitors. After 6 or 48 hours as indicated, apoptosis was assessed in the CFSE labeled L428 target cell population by flow cytometric evaluation of annexin-V binding (early apoptotic cells) or propidium iodide (PI) staining (late apoptotic cells) in the 6 and 48-hour assays, respectively (panels B and D). Control samples consisted of CFSE-labeled L428 cells alone, L428 cells alone exposed to 10 μM staurosporine (6 hour cytotoxicity assays only) and nonactivated Treg-depleted T cells (Tc) plated at a 3:1 ratio with labeled L428 target cells. Representative dot plots for a 6-hour cytotoxicity assay, with annexin-V positive apoptotic target cells highlighted with percentages, are shown in panel B. Replicates from a 48-hour cytotoxicity assays are graphed in panel D. Statistical comparison of the indicated data sets was performed using a t-test (NS-not significant with P > .05, * denotes P < .02, ** denotes P = .003). The data shown in both of the timepoints are representative of three experiments using multiple donors.

Multiple previous studies have demonstrated that Tregs expanded in rapamycin maintain their suppressive function [20,21,25]. However, such assays require co-culture of Tregs with target cells, usually CD4+ or CD8+ conventional T cells, in the presence of TCR/CD28 stimulation either via antibodies or irradiated antigen presenting cells, the very conditions that we have shown to induce granzyme B expression. In order to avoid inducing *de novo *granzyme B expression during suppression assays in Tregs previously expanded in rapamycin, and to separate the suppressive and cytotoxic function of Tregs, we developed a flow cytometry based cytotoxicity assay using a Hodgkin lymphoma cell line (L428) that we had previously observed to be susceptible to *in vitro *Treg-mediated cytotoxicity (unpublished observation). We used this observation to test the cytotoxic function of Tregs expanded *in vitro *with and without rapamycin. Peripheral blood nTregs were isolated by flow cytometry based on CD4+/CD25^bright ^characteristics (brightest 1/3 of CD25+ cells; Figure 6A/B) or by a bead-based method designed to isolate based on a CD4+, CD25+, CD127- surface antigen expression profile (Figure 6C/D). Both methods gave similar pre-expansion nTreg purities of >90%, based on FOXP3 expression. nTregs were then expanded with CD3/CD28 beads plus IL-2, with and without rapamycin (10 ng/mL) as indicated. Post-expansion Treg purities are shown in Figure 6A/C. Samples were subsequently co-cultured at a 3:1 ratio of FOXP3+ Tregs (or Treg-depleted nonactivated Tconv as a control) to CFSE-labeled L428 target cells for 6 or 48 hours as indicated. Apoptosis was measured by flow cytometric analysis of the CFSE labeled target cell fraction. Early apoptosis was measured by annexin-V binding to target cells in the 6 hour cytotoxicity assays. For the 48-hour assays, late apoptosis was measured by PI staining of the target cell population. Representative dot plots from a 6-hour cytotoxicity assay are shown in Figures 6A/B. Statistical analysis of multiple 6-hour replicates showed a significant increase in annexin-V-positive apoptotic target cells co-cultured with nTregs expanded without rapamycin (versus target cells cultured alone; P = .04). The increase in apoptotic target cells co-cultured with nTregs expanded in rapamycin was not statistically significant (P = .07). Staurosporine treated cells served as a positive apoptosis control. For the 48-hour cytotoxicity assays, representative data, including statistical significance, are shown in Figure 6C/D. Although cytotoxicity was present for both Treg subsets (+/- rapamycin), it was significantly higher for Tregs expanded without rapamycin. Thus, in this system cytotoxicity at both 6 and 48 hours correlates with granzyme B expression, and inversely with rapamycin exposure, in expanded nTregs.

## Discussion

Adoptive transfer of *in vitro *expanded, *ex vivo *peripheral blood nTregs has been suggested as a potential treatment for patients suffering from autoimmune disorders, chronic inflammatory diseases, graft-versus-host disease (GVHD) or to suppress organ rejection in transplant patients [26-28]. Indeed, early animal studies in the setting of solid organ transplantation have been promising [27,29-31]. However, the optimum conditions for generating or expanding suppressive regulatory T cells remains somewhat controversial. For the generation of suppressive, induced Tregs (iTregs) from CD4+, CD25- naïve Tconv, TGF-β1 is required [32,33] as is IL-2 [34]. For polyclonal expansion of peripheral blood nTregs, sustained T-cell receptor activation and CD28 co-receptor triggering using plate- or bead-bound anti-CD3 and anti-CD28 along with high dose IL-2 is necessary [6,35]. Interestingly, expansion of polyclonal nTregs with anti-CD3/anti-CD28 stimulation and IL-2 elevates their suppressive capabilities above those of freshly isolated nTregs in an *in vivo *murine model[6]. Thus, *in vitro *expansion may impart properties that are absent in freshly isolated nTregs. However, the mechanisms that account for this are unclear.

Tregs have an altered pattern of PI3K-mediated TCR and IL-2R signaling and, related to this, they have a unique ability to expand in the presence of rapamycin. However, other potentially unique aspects of TCR- or IL-2-mediated Treg signaling have not been thoroughly explored. Our goal was to evaluate granzyme B expression patterns in *in vitro *expanded *ex vivo *human peripheral blood nTregs and determine the signals that induce granzyme B. Furthermore, we studied the necessity for PI3K-mTOR signaling for granzyme B expression and the effects of rapamycin. We found that granzyme B was not expressed in any peripheral blood CD4+ T cell subset in the normal donors tested. Utilizing enriched samples of peripheral blood *ex vivo *nTregs typically containing approximately 70% Tregs and 30% Tconv by FOXP3 expression, we found that T cell receptor triggering alone was insufficient to induce granzyme B expression, consistent with previous reports that Tregs are anergic *in vitro *to TCR stimulation alone [36]. Similarly, high dose IL-2 (250 IU/mL) treatment alone failed to induce granzyme B expression. This observation is in contrast to previously reported findings in NK cells and CD8+ T cells where IL-2 without additional stimuli results in elevated expression of granzyme B [12,13]. We found that sustained activation of both the TCR and CD28 along with high dose IL-2, conditions optimal for Treg expansion [35], led to a rapid and marked induction of granzyme B. However, pretreatment of CD3/CD28/IL-2 expanded Tregs with rapamycin or the PI3K inhibitor LY294002 resulted in a marked suppression of granzyme B. Thus, signaling through the PI3K-mTOR pathway appears necessary to support granzyme B expression. The observation that IL-2 treatment alone fails to induce granzyme B expression may be related to the fact that Tregs, in contrast to effector T cells, do not activate downstream targets of PI3K in response to IL-2R stimulation [17] apparently due to inhibition by PTEN [37]. Thus, in addition to the lack of proliferation in response to IL-2 previously shown by others [17], the lack of granzyme B induction by IL-2 outlined here is an additional physiologic ramification of the altered IL-2R signaling in Tregs that has not previously been reported.

We, like other groups [38], found that rapamycin-treated Tregs subjected to strong CD3/CD28 stimulation in the presence of IL-2 retain their ability to proliferate. Single cell flow cytometric analysis of proliferation of Tregs and Tconv under identical expansion conditions revealed a striking difference in proliferative capacity in the setting of rapamycin treatment that appears to markedly favor Tregs. This effect was most evident at a concentration of 10 ng/mL, a dose that corresponds to therapeutic human plasma levels [23]. Higher rapamycin doses yielded suppression of both Treg and Tconv, although to a greater degree in the latter. We have also shown that the activation induced enhancement in FOXP3 protein levels in stimulated cells is similar, regardless of the presence of rapamycin or low dose PI3K inhibition. We therefore conclude that granzyme B is not simply a so-called "activation marker" but that it is subject to a distinct means of regulation that depends on a TCR/CD28 induced PI3K-mTOR mediated signaling mechanism that is distinct from those mechanisms that regulate proliferation and FOXP3 expression. Of note, many of the previous studies of the role of granzyme B in regulatory T cells have been performed in murine model systems [2-5] and studies of granzyme B in human Tregs are relatively few. Previous studies by Grossman, *et al*. utilizing human cells described expression of granzyme B in adaptive (induced) regulatory T cells generated *in vitro *by anti-CD3/anti-CD46 treatment in the presence of IL-2 that resulted in cytotoxic activity against human target cell lines [39,40]. This same group found much lower levels of granzyme B expression in CD3/CD28/IL-2 expanded natural Tregs [39]. The reason for this difference is unclear but may be related to differences in expansion conditions and strength of stimuli between this, and the current study.

We have also shown that longer term (4 days) activation results in upregulation of surface IL-7R (CD127) levels in nTregs but not in Tconv. This has the effect of equalizing post-activation CD127 expression in both T cell types. This finding indicates that using low-level CD127 expression to identify or sort nTregs may be problematic in the setting of CD3/CD28/IL-2 activated or expanded Tregs.

Induction of Foxp3 expression in Tregs differentiated *in vitro *from naïve CD4+, FOXP3- T cells treated with TGFβ is blocked by transfection with constitutively active forms of AKT [16]. However, nTregs exhibit little suppression of pre-existing FOXP3 protein levels when treated in a similar fashion [16]. Taken together, the previous data and that presented here indicate that PI3K-AKT-mTOR signaling is dispensible for nTreg proliferation, it appears to suppress *de novo *induction of FOXP3 expression in naïve T cells, it has little impact on pre-existing FOXP3 protein levels or in the activation induced increase in FOXP3 seen in activated nTregs, and it promotes a granzyme B-mediated cytotoxic function in activated nTregs. Thus, mTOR signaling may yet have a role in Treg physiology.

## Conclusion

In human cells, we confirm the finding that rapamycin treatment favors the outgrowth of nTregs in response to strong stimuli due to differential inhibition of CD4+, CD25- conventional T cells under identical expansion conditions. Furthermore, our data shows that PI3K-mTOR signaling is important for CD3/CD28 induced granzyme B expression in human nTregs. Tregs expanded in rapamycin exhibit suppressed granzyme B expression and a correspondingly lower cytotoxic activity in an *in vitro *cytotoxicity assay using a cell line as target cells. The effects of rapamycin on granzyme B expression may suggest that other suppressive strategies are dominant in rapamycin treated Tregs under physiologic conditions.

## Authors' contributions

OE performed the experiments, acquired data, analyzed data and performed statistical analysis. TK conceived of the study, performed experiments, acquired data, analyzed data, interpreted data and drafted the manuscript. All authors read and approved the manuscript.
